# A countrywide molecular survey leads to a seminal identification of the invasive cattle tick *Rhipicephalus (Boophilus) microplus* in Cameroon, a decade after it was reported in Cote d’Ivoire

**DOI:** 10.1016/j.ttbdis.2019.02.002

**Published:** 2019-04

**Authors:** Barberine A. Silatsa, Jules-Roger Kuiate, Flobert Njiokou, Gustave Simo, Jean-Marc K. Feussom, Alabi Tunrayo, Gaston S. Amzati, Bernard Bett, Richard Bishop, Naftaly Githaka, Stephen O. Opiyo, Appolinaire Djikeng, Roger Pelle

**Affiliations:** a Biosciences eastern and central Africa - International Livestock Research Institute (BecA-ILRI) hub, P.O. Box 30709-00100, Nairobi, Kenya; b Department of Biochemistry, Faculty of Sciences, University of Dschang, P.O. Box 67, Dschang, Cameroon; c Laboratory of General Biology, Faculty of Sciences, University of Yaounde I, P.O. Box 812, Yaounde, Cameroon; d Molecular Parasitology and Entomology Unit, Department of Biochemistry, Faculty of Sciences, University of Dschang, P.O. Box 67, Dschang, Cameroon; e Cameroon Epidemiosurveillance Network for Animal Diseases (RESCAM), Ministry of Livestock, Fisheries and Animal Industries, Yaounde, Cameroon; f International Institute of Tropical Agriculture (IITA), Oyo Road PMB 5320, Ibadan, Nigeria; g Department of Biosciences, International Livestock Research Institute (ILRI), Nairobi, Kenya, P.O. Box 30709-00100, Nairobi, Kenya; h Veterinary Microbiology and Pathology (VMP). Washington State University, 100 Dairy Road, 99164, Pullman, WA, USA; i Molecular and Cellular Imaging Center – Columbus, The Ohio State University, Columbus, OH, 43210, USA

**Keywords:** *Rhipicephalus microplus*, Cameroon, Genotyping, Phylogenetic tree

## Abstract

The cattle tick *Rhipicephalus microplus* is the most important arthropod vector of livestock diseases globally. Since its introduction in West Africa a decade ago, it has been reported in Ivory Coast, Benin, Togo, Mali, Burkina Faso and Nigeria with potentially far-reaching adverse impacts on the livestock sector in the region. Cameroon is located on a major route for transboundary cattle trade between Central and West Africa and it is therefore at risk from *R. microplus* invasion. This study investigated the occurrence of *R. microplus* in Cameroon, the genetic polymorphism of the tick and population structure of isolates from different regions of the country to provide data that underpin the design of future vector control programs.

A cross-sectional survey was conducted in which ticks were collected from cattle at 54 sites across the five Agroecological zones (AEZs) within Cameroon. Tick identity (sex and species) was assigned using taxonomic keys. Species identity was confirmed through amplification and sequencing of the mitochondrial COI and 16S rRNA genes. A total of 7091 ticks were collected out of which 1112 (15.6%) were morphologically identified as *R. microplus*. The presence of *R. microplus* was confirmed in 4 out of 5 agroecological zones. Only two haplotypes were identified by both COI and 16S rRNA genes, indicating a very low divergence in the genetic structure of the *R. microplus* population in Cameroon. 16S rRNA sequence analysis revealed a new haplotype specific to Cameroon. Phylogenetic trees revealed that all isolates of *R. microplus* from Cameroon were grouped into the previously described Africa/Americas clade. Application of a niche modelling algorithm to *R. microplus* distribution in Cameroon predicted that suitable habitat for the tick extended into southern Nigeria.

This study demonstrated for the first time the presence of *R. microplus* in Cameroon. Genetic diversity tests indicate that the tick has not evolved significantly since the initial introduction to West Africa. We suggest further longitudinal studies to better define the spatial and temporal expansion of the range of the tick and the drivers of this spread.

## 1 Introduction

In Sub-Sahara Africa, most farmers depend on their livestock for survival. Cattle are primarily used as source of income from the sale of meat, milk and hide and live animals. Due to current and projected population growth in developing countries, the demand for animal-derived protein is increasing considerably, making the livestock sector represent an important asset to many households (Sibhatu et al., 2015). This sector is constrained by ticks and tick-borne diseases (TTBDs) that cause significant economic losses (Jongejan and Uilenberg, 2004). A current estimate of economic losses from ticks and tick-borne diseases globally is approximately US$20-30 billion per annum (Lew-Tabor and Rodriguez Valle, 2016). It is very difficult to measure accurately the losses due to TTBDs in Africa, however, one study in Eastern Africa using a simple spreadsheet model have estimated at about US$ 364 million the total annual losses resulting from production losses, treatment and control costs associated with tick-borne diseases (TBD) in Tanzania alone (Kivaria, 2006). In Brazil and Australia, *R. microplus* could lead to annual economic losses of about US$ 3.24 billion and US$ 62 million, respectively (Grisi et al., 2014; Singh et al., 2014).

*R. microplus* is the most important ectoparasite and disease vector of livestock globally. It transmits several pathogens including *Babesia bigemina*, *B. bovis* and *Anaplasma marginale* (Guerrero et al., 2006). Its notorious character is attributed not only to its competency to transmit a broad range of parasites, but also its ability to develop resistance against acaricides (de la Fuente et al., 2007; Howard, 1908; Jongejan and Uilenberg, 2004; Li et al., 2007; Lynen et al., 2008; Madder et al., 2011; Rodriguez-Vivas et al., 2011). Moreover, this tick has the ability to invade and displace endemic species of the same genus (De Clercq et al., 2012; Madder et al., 2012).

*R. microplus* is distributed in tropical and subtropical areas including India, Malaysia, China, Central and South-America and Australia where it has been present for decades (Cutulle et al., 2009; Estrada-Pena and Venzal, 2006; Evans et al., 2000; Labruna et al., 2009; Low et al., 2015). The dreaded tick was introduced in Madagascar towards the end of nineteenth century following the importation of cattle from India. It subsequently moved to Southern and Eastern Africa (Berkvens et al., 1998; Lynen et al., 2008; Macleod and Mwanaumo, 2009; Nyangiwe et al., 2013; Smeenk et al., 2000; Tonnesen et al., 2004; Walker et al., 2003; Wedderburn et al., 1991). In West Africa, *R. microplus* was first reported in Ivory Coast in 2007 (Madder et al., 2007). Subsequently, the tick was reported in Benin and is believed to have been introduced through the importation of Gir and Girolando cattle breeds from Brazil.

The tick has rapidly spread in the sub-region, probably as a result of transhumance of cattle (Adakal et al., 2013; De Clercq et al., 2012; Madder et al., 2012, 2011; Madder et al., 2007). Most recently, the presence of the tick was confirmed using molecular tools in the Western Nigeria region bordering Benin. (Kamani et al., 2017). Despite the number of studies reporting *R. microplus* in several countries of different African sub-regions, the current situation remains unknown in Central Africa.

In Cameroon for instance few studies have been undertaken to identify ticks infesting cattle. For these studies, morphological methods have been used for the identification of different tick species (Awa, 1997; Awa et al., 2015; Bayemi, 1991; Mamoudou et al., 2016; Merlin et al., 1986, 1987; Ndi et al., 1991). However, the identification of *Rhipicephalus (Boophilus)* spp. using morphological criteria is challenging because the size and the morphological differences between *R. annulatus*, *R. decoloratus*, *R. geigyi*, *R. kohlsi* and *R. microplus* is very limited and variable (Lempereur et al., 2010). These challenges can be resolved using molecular markers for tick identification and for the inference of phylogenetic relationships between closely related tick lineages.

One major risk factor for the spread of livestock diseases and their vectors is the movement of live animals. While registration and tracing systems have been implemented in developed countries in order to set up early warning systems for outbreaks detection and control, in most low and middle income countries including Cameroon, there are no systematic records on animal movement (Todaro and Smith, 2014). Because of its strategic location in the Central Africa region, Cameroon plays a pivotal role in livestock trade both within the region, and between Central Africa and West Africa. Beside the movement of animals for trade purposes, transhumant pastoralism is a regional phenomenon in West and Central Africa. Live animals move from West Africa to Cameroon especially during the dry season. These uncontrolled animal movements across borders render Cameroon epidemiologically connected with both neighbouring and non-neighbouring West African countries (Kamuanga et al., 2008; Mian-Oudanang and Duteurtre, 2013; Motta et al., 2017; Njoya et al., 2003). Cameroon displays a great diversity of agro-ecological zones (AEZs) and has an equatorial climate suitable for the establishment of *R. microplus* (De Clercq et al., 2015). Therefore, the risk of introduction and expansion of this efficient tick vector in Cameroon is high and requires regular surveillance. Accurate identification of the tick is a critical step towards surveillance and control of ticks and tick borne-diseases transmission. In addition to classical methods based on morphological criteria, molecular approaches targeting mitochondrial Cytochrome Oxidase I (COI) and 16S ribosomal ribonucleic acid (16S rRNA) genes have been widely used in the molecular characterisation and phylogenetic studies of *R. microplus* populations. The successful application of the mitochondrial COI and 16S rRNA genes sequences in resolving the phylogenetic relationship within the *R. microplus* complex has been demonstrated (Burger et al., 2014; Csordas et al., 2016; Low et al., 2015). These molecular markers have provided data that has enabled the resolution of the taxonomic status of tick species, the spatial limits of populations, and the nature of gene flow among tick’ populations, in addition to increased understanding of the epidemiology of the diseases they transmit.

This study aimed to clarify the status of *R. microplus* in Cameroon and its distribution across all five agro-ecological zones (AEZs) that characterize the country. Additionally, the study investigated the genetic polymorphism and population structure of the *R. microplus* isolates collected across the country. Collectively this data will be important in underpinning future vector control programs.

## 2 Materials and methods

### 2.1 Study area

**Fig. 1:**
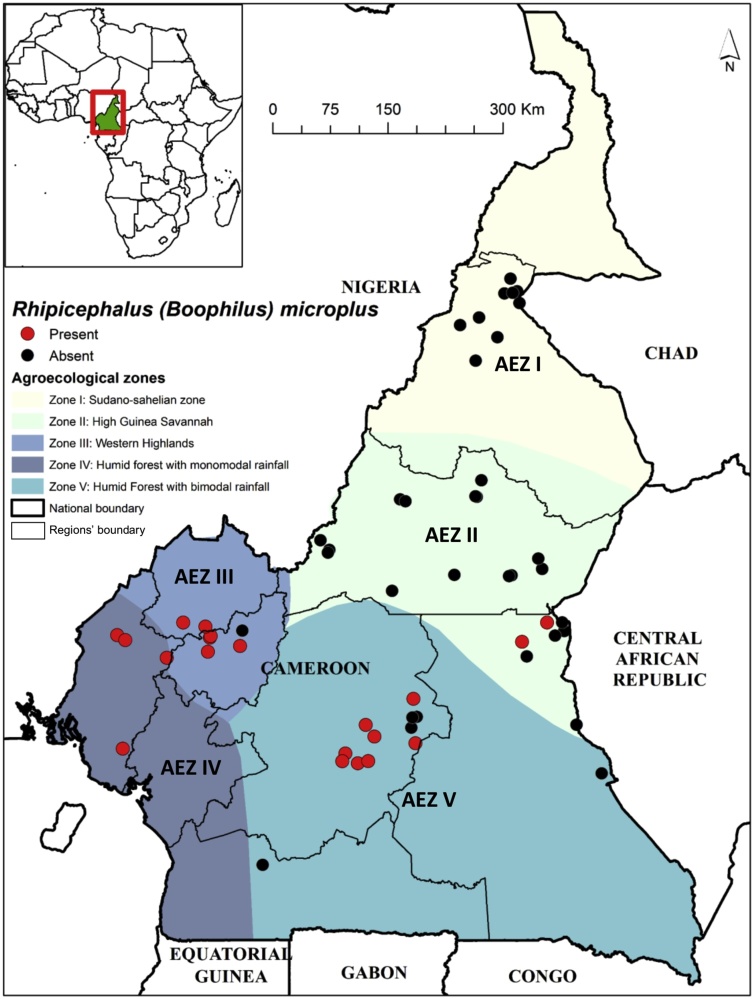
Map showing sampling sites as well as locations where *R. microplus* species have been identified in Cameroon.

Cameroon is located in Central Africa region and extends from 2° to 13 °N latitude and between 9°–16 °E longitude. Because of its geographic position and climatic variations, Cameroon exhibits all major climates and vegetation of the continent (coast, desert, mountains, rainforest, and savannah). Thus, Cameroon is divided into five major agro-ecological zones (Fig. 1). The Sudano-Sahelian zone (AEZ I) is situated at 250–500 m above the sea level and is characterised by dry savannah and steppes. The rainy season lasts three months (June to August) and the annual precipitations range between 650–1000 mm with an annual average temperature of 28.9 °C. The High Guinea Savannah zone (AEZ II) is situated at 500–1500 m above the sea level and is characterized by savannah degraded forest. The rainy season extend from April to October and the annual precipitations between 1500 to 1800 mm with annual average temperature of 22.06 °C. The Western Highlands zone (AEZ III) is located in the mid and high-altitude zone of the country. The vegetation is characterized by savannah and degraded forest. The annual average temperature is 20.64 °C and the annual precipitations range from 1300 to 3000. The rainy season last 8 months (March to October). Its agro-climatic conditions are favourable for cattle rearing and that explains why this zone together with AEZ I and II constitute the most important cattle production areas in Cameroon. The zone IV or the Humid Forest zone with mono-modal rainfall is found to the west of the south Cameroon plateau. It has an evergreen forest. The wet season last eight months, from March/April to October/November. The annual rainfall ranges from 3000 to 4000 mm and the average temperature is between 23 °C and 28 °C. This zone is situated between 0 to 2500 m above the sea level. The Humid Forest zone with bimodal rainfall (AEZ V) lies at altitude ranging between 400 and 1000 m above the sea level. This zone is characterized by a humid forest and savannah mosaic. The average temperature is about 26 °C and the annual average rainfall is 2456.8 mm.

The majority of participants were traditional smallholder farmers practising small-scale animal husbandry. Sheep and goats are usually reared together with cattle. The grazing system is essentially free grazing and to a lesser extent a combination between free grazing and stall-feeding. Few ranches were sampled and in areas where farms were not accessible, markets and slaughterhouses were targeted.

### 2.2 Ticks sampling and morphological identification

The countrywide cross-sectional survey took place between April and August 2016 in all five AEZs (Fig. 1). A total of 54 sites where sampled. All the body parts of the cattle were examined. Using forceps, ticks were plucked directly from the cow skin to preserve the mouthparts and placed in tubes containing 70% ethanol. Afterwards, ticks were transported to the Tick Unit at the International Livestock Research Institute in Nairobi for identification. The identification of sex and species of each tick was carried out on a stereoscopic microscope using the taxonomy keys as previously described by Walker et al. (2003).

### 2.3 Genomic DNA extraction

Prior to the extraction of genomic DNA, adult ticks were washed twice in distilled water and air dried for 30 min. Individual ticks were transferred into 2 ml sterile Eppendorf tubes containing one sterile glass bead and placed in liquid nitrogen for 5 min. Tick was then thoroughly grinded to powder using a Genogrinder (SPEX Sample Prep). Genomic DNA was extracted using the DNeasy Blood & Tissue Kit (Qiagen, Germany) as recommended by the manufacturer. The integrity of extracted DNA was checked on a 1% agarose gel electrophoresis. The final concentration of DNA was determined with a spectrophotometer (WPA Lightwave II, Biochrom, UK).

### 2.4 PCR amplification and amplicon sequencing

To confirm the identity assigned to *R. microplus* using morphological traits and to subsequently study its population structure, we carried out molecular analysis of mitochondrial COI and 16S rRNA gene sequences. For the COI gene, a 700 base pairs (bp) PCR fragment was generated using the forward COXI.2 F (5′-CTTCAGCCATTTTACCGCGA-3′) and reverse COX-I.2R (5′-CTCCGCCTGAAGGGTCAAA-3′) primers as previously described (Csordas et al., 2016). The primers 16S-F (5′-TTAAATTGCTGTRGTATT-3′) and (16S-R1 5′-CCGGTCTGAACTCASAWC-3′) previously described were used for the 16S rRNA gene and the expected PCR amplicon was 455 bp (Lv et al., 2014). For both gene, PCR was performed in 50 μl reaction comprising 25 μl AccuPower® Taq 2x PCR Master Mix (Bioneer, Korea), 50 ng of DNA template, 0.2 μM of each primer. The final volume of the reaction mixture was completed to 50 μl with nuclease free distilled water. The PCR protocol for COI was as follows: an initial denaturation step at 94 °C for 3 min; followed by 35 cycles of 94 °C for 1 min, 56 °C for 30 s, 68 °C for 1 min; and a final extension step of 68 °C for 7 min. The protocol for 16S rRNA was as follows: an initial denaturation step 94 °C for 3 min; followed by 5 cycles of 94 °C for 30 s, 49 °C for 30 s, and 68 °C for 30 s; 5 cycles of 94 °C for 30 s, 47 °C for 30 s, and 68 °C for 30 s; 5 cycles of 94 °C for 30 s, 45 °C for 30 s, and 68 °C for 30 s; 25 cycles of 94 °C for 30 s, 43 °C for 30 s, and 68 °C for 30 s; followed by a final extension step of 68 °C for 5 min. 5 μl of the PCR product was run on a 1.8% agarose gel in order to check the quality of the amplicons. The amplified COI and 16S rRNA gene fragments were purified using the QIAquick PCR Purification Kit (Qiagen, Germany) following the manufacturers’ guide. The purified amplicons of COI and 16S rRNA genes were sequenced at Bioneer (Korea), using the same gene specific forward and reverse primers pair used for PCR amplification.

### 2.5 Data analysis

#### 2.5.1 Sequences analysis

The two chromatograms of each individual (forward and reverse sequences) amplicon were edited manually and a consensus sequence was generated. For each gene, 76 individual sequences were aligned using CLC Main Workbench software v7.8.1 (CLC bio, Aarhus, Denmark). To confirm the identity of each species of tick, its sequences were compared with those available in the GenBank by Blast (https://blast.ncbi.nlm.nih.gov/Blast.cgi).

#### 2.5.2 Genetic diversity and phylogenetic analysis

Sequences from the multiple sequence alignment were extracted and collapsed into haplotypes using DnaSP software v5.10.01 (Librado and Rozas, 2009). A population was delimitated by an AEZ. For each and for the overall population, Haplotype diversity (Hd), Nucleotide diversity (pi), number of haplotypes (h) values were determined using Arlequin v3.5.2.2 (Excoffier and Lischer, 2010) and DnaSP software v5.10.01. All representative haplotypes (references) of *R. microplus* across the world were retrieved from the National Center for Biotechnology Information (NCBI) database (https://www.ncbi.nlm.nih.gov) (Table 1).

**Table 1:** *R. microplus* COI and 16S rRNA haplotype gene sequences retrieved from the GenBank.

Gene locus	Geographical origin	Number of sequences	GenBank accession number	Reference
**COI**	Benin	1	KY678120	(Duron et al., 2017)
	Brazil	7	KP226159-60; KP226168-69; KP226174	Direct submission
			KC503261; NC_023335	(Burger et al.,2014)
	Cambodia	1	KC503260	(Burger et al.,2014)
	China	7	JQ737082-83; KU664523	Direct submission
			KC503259	(Burger et al.,2014)
			KF583579; JX051125; JX051119	(Lv et al., 2014)
	Colombia	4	KT906177-78; KT906180-81	Direct submission
	India	9	KP698515-16; KP792578; KP792586-87; KP318133	Direct submission
			KX228541; KX228543-44	(Goolsby et al., 2016)
	Kenya	1	KX228549	(Goolsby et al., 2016)
	Madagascar	1	KY678118	(Duron et al., 2017)
	Malaysia	8	KM246866-68; KM246870-72; KM246875-76	(Low et al., 2015)
	Pakistan	1	KY373260	(Rehman et al., 2017)
	Panama	1	KF200106	Direct submission
	Philippines	2	KX228545; KX228548	(Goolsby et al., 2016)
	South Africa	1	KY678117	(Duron et al., 2017)
	USA	1	KP143546	(McCooke et al., 2015)
**16S rRNA**	Argentina	1	EU918176	Direct submission
	Bolivia	1	EU918177	Direct submission
	Brazil	1	EU918178	Direct submission
	China	8	KU664517	Direct submission
			JX051062-63; JX051068; JX051072	(Lv et al., 2014)
			JF979381	(Chen et al., 2012)
			KX450285; KJ652224	Direct submission
	Costa Rica	1	EU918179	Direct submission
	India	17	HM536970-71, HM536977; GU722605;	Direct submission
			EU918188; GU817006; HM536976; JF927707	
			JN979989; KP210052; KY458969; KC953868;	
			JX974347; KP210055; KP210049	
			GU222462; GU323288	(Kumar et al., 2011)
	Japan	1	AB819268	Direct submission
	Malaysia	6	KM246879-84	(Low et al., 2015)
	Mozambique	1	EU918187	Direct submission
	Paraguay	1	EU918180	Direct submission
	Peru	1	EU918181	Direct submission
	South Africa	1	EU918182	Direct submission
	Taiwan	2	AY974232; AY974241	Direct submission
	Tanzania	1	EU918183	Direct submission
	Thailand	2	KC170742; KT428015	Direct submission
	Uruguay	1	EU918184	Direct submission
	USA	1	L34310	(Black and Piesman, 1994)

To determine the relationships between *R. microplus* populations and infer their evolutionary history, the haplotypes generated were compared to the reference sequences available in the GenBank. The pattern of DNA sequence polymorphism along COI and 16S rRNA gene fragments was generated (Tables S1 and S2). The phylogenetic reconstruction was performed on COI and 16S rRNA gene sequences. The best nucleotide substitution model was identified and the Neighbour-Joining (NJ) and/or Maximum Likelihood (ML) trees were plotted using 1000 bootstrap replicates in MEGA v7.0. The out-groups for both genes were *R. decoloratus* (AF132826 (COI) and EU918193 (16S rRNA)), *R. geigyi* (KC503263 (COI) and KF569942 (16S rRNA)) and *Dermacentor nitens* (KF200099 (COI) and KC503258 (16S rRNA)).

#### 2.5.3 Niche modelling for *R. microplus*

The random forest (RF) algorithm in R (version 3.1.1) was used to analyse spatial distribution of the tick in Cameroon. The same model was also used to predict the tick’s suitable habitats in Nigeria. The model used Version 2.0 of bioclimatic data obtained from the WorldClim – Global Climate Data (http://www.worldclim.org/bioclim, accessed on 17th March 2018) as predictor variables. These data were downloaded as raster files with a resolution of 5 min (Fick and Hijmans, 2017). They were then clipped using country shapefiles and coordinates of the sampling points used to extract values of the predictor variables for fitting the model. The model used a total of 550 trees and the number of variables used at each split were 6. The importance of each of the 19 bioclimatic variables was assessed by examining mean increase/decrease in classification accuracy (mean square error) when that variable was removed. To analyse the fit of the model, data were split into training set (comprising 75% of the data) and model testing set (25% of the data).

## 3 Results

### 3.1 Morphological identification and distribution range of *R. microplus*

A total of 7091 ticks were collected in 54 sites across all five AEZs of Cameroon. On the basis of morphological criteria, three genera of ticks were identified: *Amblyomma* spp. was the most abundant and represents 59% (4210/7091) of the collected ticks, followed by *Rhipicephalus* spp. (27%) and *Hyalomma* spp. (14%). For this study, the focus was only on *R.s microplus* data while the analysis of the other tick species will be reported elsewhere. Out of 7091 collected ticks, 1112 (15.6%) were identified morphologically as *R. microplus*. This species was found in 20 out of 54 collection sites (Table 2). *R. microplus* was present in the AEZs II, III, IV and V meanwhile it was absent in AEZ I that covers the North and Far-North regions of Cameroon. The distribution map clearly shows that *R. microplus* distribution range follows climatic patterns. The tick was present mainly in the southern equatorial part of the country characterized by low temperature and high rainfall (Fig. 1).

**Table 2:** Prevalence and abundance of *R. microplus* per AEZs.

AEZ	Total no. of sampling sites	No. of sites infested by *R. microplus*	Prevalence of *R. microplus (%)*	No. of ticks (all species)	No. of *R. microplus* ticks	Relative abundance of *R. microplus* (%)
I	9	0	0.0	1667	0	0.0
II	20	2	10.0	2834	5	0.2
III	8	7	87.5	564	288	51
IV	3	3	100.0	158	103	65.2
V	14	8	57.1	1868	716	38.3
Overall	54	20	37.0	7091	1112	15.6

### 3.2 Confirmation of morphological identification by molecular tools

For each sampling site, the identification of *R. microplus* with morphological tools was confirmed using both COI and 16S rRNA molecular markers. COI and 16S rRNA sequences were generated from 76 tick specimens randomly selected in the AEZs II, III, IV and V where the *R. microplus* tick was collected. The final sequence size for COI and 16S rRNA gene fragments were respectively 600 bp and 409 bp. BLAST analysis of these sequences revealed a high identity value with the sequences of *R. microplus* available in the GenBank database. For each sampling site, results of both COI and 16S rRNA genes confirmed the morphological identification of *R. microplus,* highlighting thus the presence of this tick species in Cameroon. For the 76 COI sequences analysed, Blast identity value was 100% *R. microplus* whereas for 76 16S rRNA sequences, it ranged from 99% to 100% (Table 3).

**Table 3:** BLAST results for sequences of the COI and 16S rRNA gene fragments.

Gene locus	Haplotype	Blast results				
		E value	Identity %	Accession number	Origin	Reference
COI	H1	0	100	KY678120	Benin	(Duron et al., 2017)
	H2	0	100	KP226169	Brazil	Direct submission
16S rRNA	Hp1	0	100	KC503261	Brazil	(Burger et al., 2014)
	Hp2	0	99	KP143546	USA-Texas	(McCooke et al., 2015)

### 3.3 Haplotype and nucleotide diversity

**Fig. 2:**
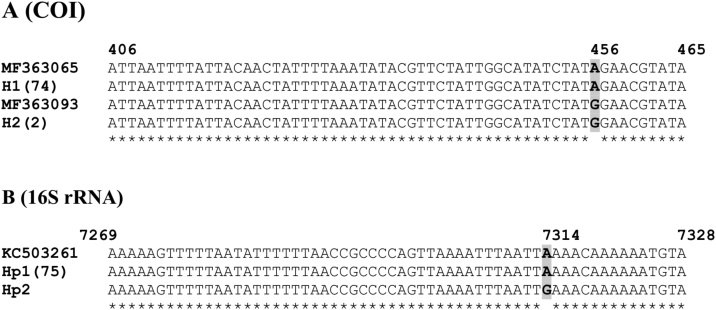
Multiple partial nucleotide sequence alignment of the (A) COI and (B) 16S rRNA genes haplotypes obtained in this study. Only the regions containing the identified SNPs, in bold, are shown. Haplotypes are H1 and H2 for COI and Hp1 and Hp2 16S rRNA. Residue coordinates are listed above the alignment and the positions of the polymorphic residues are indicated. The GenBank accession No. of the reference sequences used are indicated. The frequency of each haplotype is indicated in square brackets, when larger than 1. Identical residues in all sequences are identified below the alignment (*).

For COI gene, the overall sequences analysis reveals Haplotype diversity (Hd) and Nucleotide diversity (pi) of 0.052 and 0.00009 respectively. These values were very low. The overall data set of COI showed only one single nucleotide polymorphic site A/G at position 504, with reference to the COI sequence, Acc. No. KY678120, (Fig. 2A) that was parsimony informative (each of these mutations occurs in at least two of the sequences). All the sequences yielded two haplotypes, H1 and H2 with 97% and 3% frequency, respectively. H1 was widely spread in the 4 AEZs while H2, represented by the tick samples III156 and III48 was only present in the AEZ III.

For the 16S rRNA gene, the Haplotype diversity and Nucleotide diversity values were 0.026 and 0.00006 respectively. As for COI, these values were very low. The analysis of 16S sequence reveals one new mutation (A was replaced by G), which was singleton (G occurred in only one sequence: 16SIII64) at position 7314, with respect to the *R. microplus* isolate BomiB mitochondrion, complete genome, GenBank Acc. No. KC503261 (Fig. 2B). Therefore, for this gene marker, two haplotypes were observed: Hp1 which was the most frequent (99%) and the most ubiquitous and Hp2 that was represented by only one sequence from the tick sample III64 found in the AEZ III. This haplotype was new and appears unique to Cameroon. For both COI and 16S rRNA genes, sequences analysis reveals similar results, as each one identified a major and a minor haplotype. Nevertheless, while the major haplotypes contained practically the same ticks, the single tick in the 16S minor haplotype was different from the two forming the COI minor haplotype. Thus, all in all, both COI and 16S rRNA gene analysis identified 3 haplotypes in total; the major haplotype was randomly distributed whereas the two minor haplotypes were found only in the AEZ III (Table 4). The new haplotype gene sequence identified from this study was deposited in the NCBI GenBank (an accession number has not yet been released).

**Table 4:** *R. microplus* COI and 16S rRNA haplotypes distribution and genetic diversity indices in Cameroon.

AEZ	N	COI				16S rRNA			
		h	Haplotypes (frequency %)	pi (SD)	Hd (SD)	h	Haplotypes (frequency %)	pi (SD)	Hd (SD)
II	4	1	H1(100)	0	0	1	Hp1(100)	0	0
III	34	2	H1(94), H2(6)	0.00019(0.00012)	0.114(0.071)	2	Hp1(97), Hp2(3)	0.00014(0.00013)	0.059(0.055)
IV	16	1	H1 (100)	0	0	1	Hp1(100)	0	0
V	22	1	H1(100)	0	0	1	Hp1(100)	0	0
Overall	76	2	H1(97), H2(3)	0.00009(0.00006)	0.052(0.035)	2	Hp1(99), Hp2(1)	0.00006(0.00006)	0.026(0.025)

### 3.4 Phylogenetic analysis

For phylogenetic analysis, haplotypes from this study were compared with *R. microplus* gene sequences from other countries. Forty-five sequences of COI and 47 sequences of 16S RNA were retrieved from GenBank. Neighbor Joining (NJ) and Maximum Likelihood (ML) trees analyses showed the same topology but with different bootstrap support values. All COI sequences were trimmed at 394 bp in order to obtain fragment with the same length while keeping the polymorphic site previously identified. From the 47 sequences (45 from GenBank and 2 haplotypes from this study), 23 COI haplotypes were identified. The most widely distributed were mH2 and mH4. Haplotype mH2 was found in Cameroon, Columbia, Panama, USA and Brazil while mH4 was shared by Cameroon, Madagascar, South Africa, Benin and Brazil. The distribution of the different haplotypes is shown in Table S1. The 49 sequences (47 from GenBank and 2 haplotypes from this study) of the 16S rRNA gene were collapsed into 22 haplotypes among which haplotypes mHp4 is specific to Cameroon (Table S2). This suggests that this strain of *R. microplus* identified is specific to Cameroon

**Fig. 3:**
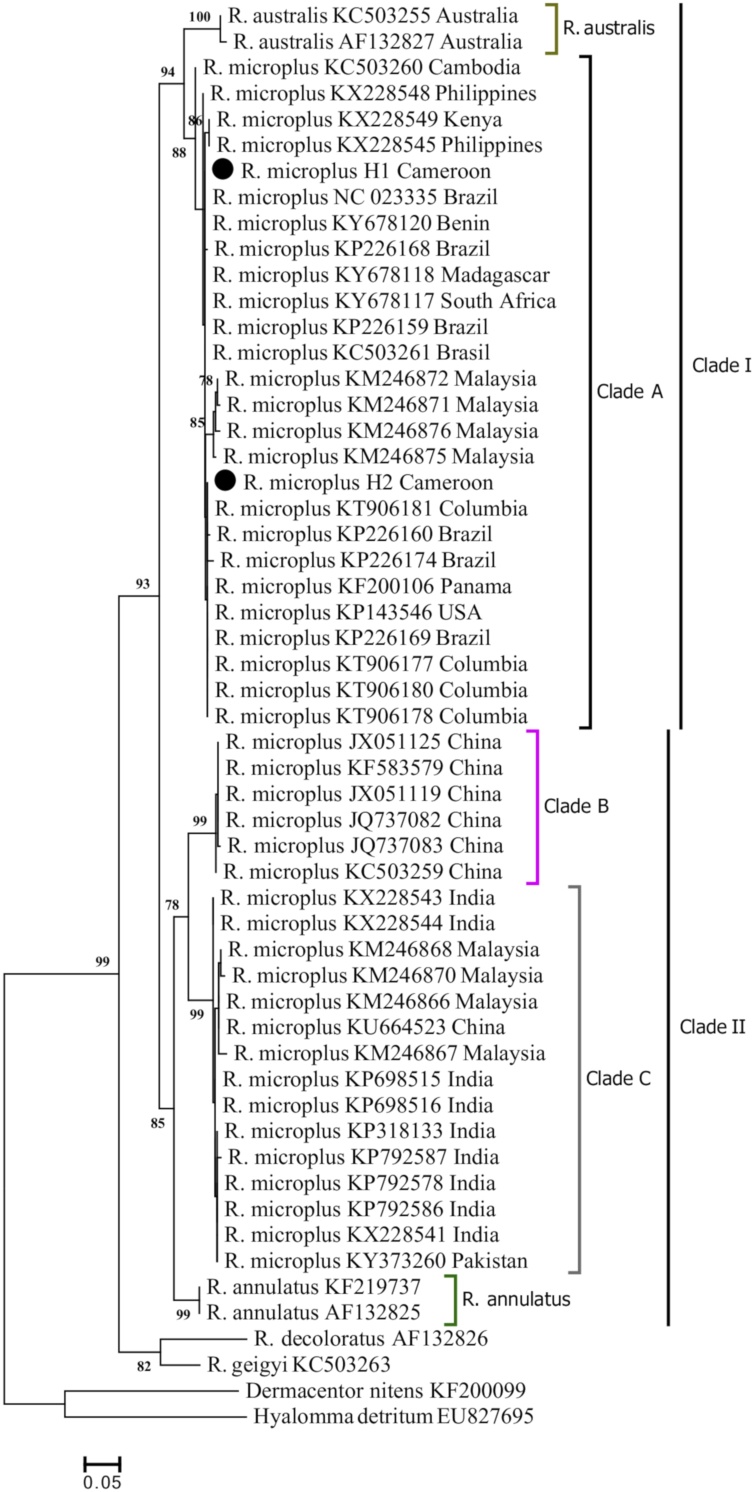
Molecular phylogenetic analysis of COI gene sequences of *R. microplus* by Maximum likelihood method. The evolutionary history was inferred by using Hasegawa-kishino-yano model. A discrete Gamma distribution was used to model evolutionary rate differences among sites (5 categories (+G, parameter = 0.3885)). The percentage of trees in which the associated taxa clustered together is shown next to the branch. Specimens sequenced in this study are highlighted.

**Fig. 4:**
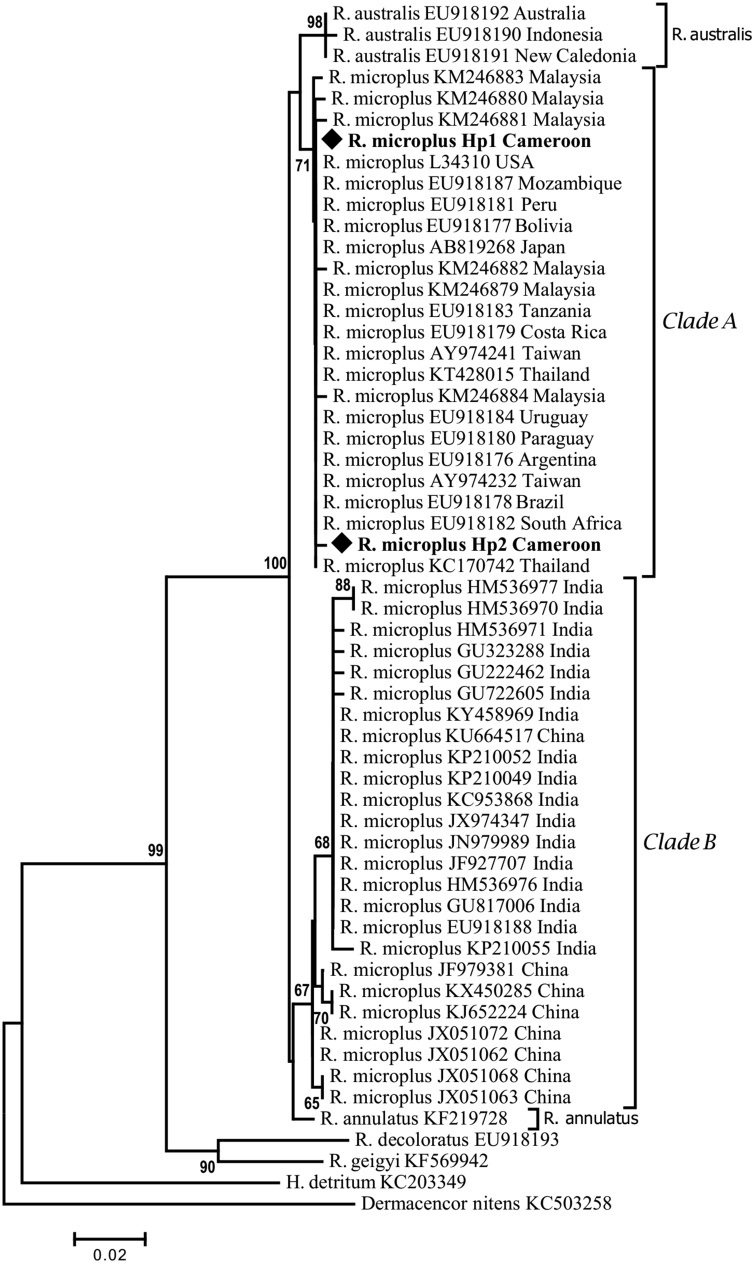
Neighbour-Joining tree of 16S rRNA haplotype gene sequences for *R. microplus* across the world. The evolutionary distances were computed using the Tamura 3-parameter method. Bootstrap values (>63) are displayed above nodes. Specimens sequenced in this study are in bold type.

The COI ML phylogenetic tree revealed two major genetic clades supported with a strong bootstrap value of 93%. Clade I comprises the previously described clade A sensu by Burger et al. (2014) which showed a sister relationship with the *R. australis* clade. Clade II was divided into 3 sister clades: clade B previously described by Burger et al. (2014), clade C described by Low et al. (2015) and *R. annulatus* clade. The 2 haplotypes (H1 and H2) generated from this study are both present in the clade A sensu (Burger et al., 2014) (Fig. 3). The phylogeny relationship inferred from 16S RNA gene sequences showed 2 principal clades well supported by a NJ bootstrap value of 100%. Clade A is sister to *R. australis* whereas clade B is sister to *R. annulatus*. The two haplotypes to which belong the samples analysed in this study are clustered in clade A (Fig. 4).

### 3.5 Niche modelling for *R. microplus*

**Fig. 5:**
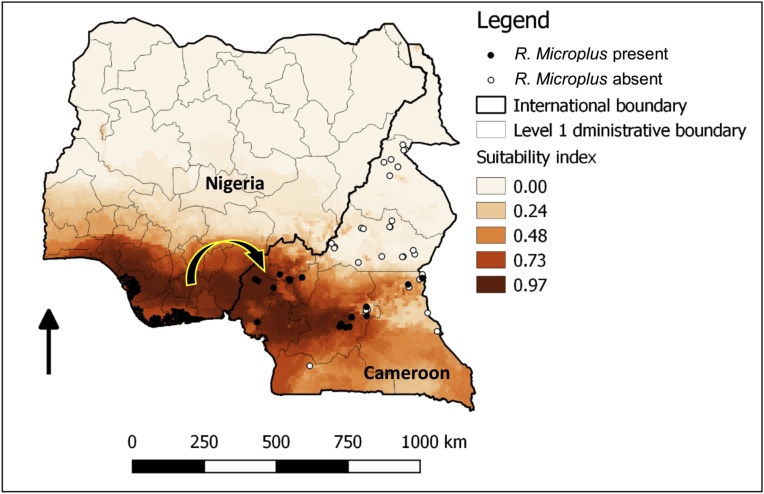
Estimated geographical distribution of *R. microplus* in Cameroon based on bioclimatic variables from WorldClim. Suitable habitats for *R. microplus* in Cameroon and Nigeria determined from ecological niche modelling. The distribution of *R. microplus* in Cameroon are from this study.

Two variables, monthly mean diurnal range in temperature (i.e., maximum temperature – minimum temperature) and annual precipitation are important in determining the spatial ranges of *R. microplus*. Suitable habitats have mean monthly diurnal range of less than 8 °C, and an annual precipitation of greater than 2000 mm. Fig. 5 shows outputs of *R. microplus* distribution modelling based on bioclimatic variables from WorldClim in Cameroon and Cameroon and Nigeria, respectively. The niche modelling results for *R. microplus* distribution in Cameroon predicts an extension of the suitable habitat for the tick into the southern region of Nigeria (Fig. 5).

## 4 Discussion

*R. microplus* is exotic to Africa. Its introduction and invasion have been well described in Western, Southern and Eastern Africa. The current situation in the Central African region including Cameroon is currently not well understood. We hypothesize that following the recent introduction of *R. microplus* from Brazil into West Africa, uncontrolled live animal movements in the region might have resulted in the introduction and spread of this highly efficient vector of multiple pathogen species in Cameroon. The cross-sectional countrywide study described herein was designed to investigate whether *R. microplus* is present in Cameroon and map its current distribution. To improve our knowledge of *R. microplus* in Cameroon, the study also analysed the intraspecific variation of mitochondrial DNA to better understand its genetic structure and its dispersal pattern.

The tick was first identified using morphotaxonomy keys. Subsequently, molecular techniques were applied to refine and validate the results of the morphological identification. This study reports for the first time, the presence of *R. microplus* in Cameroon. These results reveal for the first time, the presence of this tick species in Central Africa as already reported from other African sub-regions. *R. microplus* was exclusively abundant in the southern part of the country where the rainfalls levels are high. High annual rainfall is required for the establishment and persistence of *R. microplus* populations (Estrada-Pena and Venzal, 2006). This distribution is in line with recent predictive models describing areas featuring an equatorial climate as highly suitable for the establishment of *R. microplus* (De Clercq et al., 2015). No *R. microplus* was recorded in the Adamaoua (comprising the major part of AEZ II) and the North (part of AEZ I) regions despite the fact that more than half of total number of ticks was collected in these areas. These findings are in accordance with both recent and earlier studies that did not find *R. microplus* in the Adamaoua region (Awa, 1997; Awa et al., 2015; Mamoudou et al., 2016) and the North region (Awa, 1997). Nevertheless, we identified five *R. microplus* specimens in the northern part of the eastern region (AEZ II), probably as a result of rapid dissemination of the tick through the cattle trade network (Motta et al., 2017). Furthermore, we also identified *R. microplus* in the northwest region, contrary to Awa et al. (2015). The low number of individuals identified in AEZ II couple with the total absence of *R. microplus* in the northern AEZ I seems to indicate that climatic conditions in this part of Cameroon are not suitable for the establishment of a viable tick population in those areas. This is in accordance with a recent study using two different modelling approaches with different datasets and prediction variables that suggested the existence of a northern limit of the potential climate suitability zone for *R. microplus* in West Africa (De Clercq et al., 2013). If the northern limit of distribution observed in West Africa was extrapolated to Central Africa, the present distribution of *R. microplus* in different AEZs of Cameroon is entirely consistent with the modelling approaches. However, the models need to be interpreted with caution, because *R. microplus* will likely change its geographic range in the coming decades in response to climatic changes resulting from global warming and changes in rainfall (Lynen et al., 2008; Ogden and Lindsay, 2016). Lynen et al. (2008) has indeed described a shift in *R. microplus* population in Tanzania. The dreaded tick has extended its distribution and is now present in all the northern regions of Tanzania that were previously thought to be out of its range.

Motta et al. (2017) analysed cattle trade network within Cameroon and across the borders. The authors characterized the cattle trade network in Cameroon as a “small world” network which is favorable to the spread of vectors and their associated pathogens. This could explain why the tick, after its introduction, has rapidly spread to four out of five AEZs in Cameroon. One possible route of introduction of *R. microplus* in Cameroon is cattle movement from Nigeria due to cross-border transhumance since a recent study has confirmed the presence of *R. microplus* in Nigeria (Kamani et al., 2017).

Over the last decade, the mitochondrial COI and 16S rRNA genes have been used to resolve the phylogenetic relationships between *R. microplus* and other species of ticks such as *R. australis* and *R. annulatus.* (Burger et al., 2014; Csordas et al., 2016; Low et al., 2015). In this study, analysis of COI and 16S rRNA gene sequences revealed two haplotypes for each, with the major haplotypes being similar and the minor haplotypes being different, in the overall population of *R. microplus* in Cameroon. The study also revealed low haplotype diversity and low nucleotide diversity within *R. microplus* populations in Cameroon. This low genetic variability supports the hypothesis that *R. microplus* population in Cameroon is derived from a founder event that accompanied the recent introduction of the tick into West Africa a decade ago (Madder et al., 2007). The initial founder individuals imported from Brazil have probably colonised the West Africa region and formed a new population in Cameroon, within which limited genetic diversity is observed. Similar findings have been observed in the *R. microplus* population of New South Wales (Cutulle et al., 2009).

The low differentiation in the genetic structure in *R. microplus* populations in Cameroon suggests that *R. microplus* populations in the country share an undifferentiated gene pool. *R. microplus* is a single host tick which feeds preferentially on cattle. Therefore, movements of cattle (along with the ticks they carry) for trade purposes or as a result of transhumance have facilitated rapid dissemination of ticks in four AEZs resulting in reduced genetic variation between geographically distant populations (Kanduma et al., 2016; Sattler et al., 1986).

For both COI and 16S rRNA gene sequences, the phylogenetic analysis demonstrated that the three haplotypes described in this study are clustered within a monophyletic group. This suggests that a monophyletic lineage of *R. microplus* invaded Cameroon as previously described in Chennai (Veeramani et al., 2014).

The phylogenetic analysis of 16S rRNA and COI sequences generated trees that are consistent with previous studies (Burger et al., 2014; Low et al., 2015). These phylogenetic analyses reveal that *R. microplus* population from Cameroon has the same genetic lineage with homologues from Benin and Brazil. Furthermore, ticks from Brazil and Benin show a pattern of COI sequence polymorphism very similar to that observed in Cameroon (Table S1), implying that they have been affected by the same evolutionary process. This finding is consistent with the hypothesis that the *R. microplus* population present in Cameroon might have a Brazilian origin, and that uncontrolled animal movement in the West and Central Africa region could have played an important role in the introduction of this tick from Benin to Cameroon via Nigeria. This is consistent with our niche modelling for *R. microplus* distribution in Cameroon that predicted suitable habitat for the tick across Nigeria and Cameroon. The phylogenic relationships inferred here may not be precise because the number of sequences found in GenBank was very limited for some countries (for example there is only one COI sequence from West Africa) and some of the sequences retrieved were very short and therefore not included in the analysis. However, the findings from this study provide a basis that will inform and underpin future tick vector control programs in Cameroon. From a vector control perspective, if a biological control was to be envisaged, our data do not show any subdivision in the populations of *R. microplus* present in Cameroon that could limits its effectiveness.

The recognition of *R. microplus* as the most economically important tick species worldwide is attributed to several factors including its ability to rapidly develop resistance to major chemical classes of acaricides (de la Fuente et al., 2007). The major component of integrated tick control methods in Cameroon is the use of acaricides (Abah et al., 2017). Therefore, further studies are required to understand the status and magnitude of acaricide resistance in the country. *R. microplus* transmits the protozoan blood parasite *Babesia* that causes babesiosis in infected cattle, and which is a major cause of reduced livestock production. A future study is envisaged to assess the presence and prevalence of tick-borne pathogens associated with *R. microplus* in Cameroon.

## 5 Conclusion

This study revealed for the first time that *R. microplus* has become established in Cameroon and provided initial insights into the genetic structure of this tick in Central Africa. The study highlighted a low genetic variation in *R. microplus* population in Cameroon. We speculate that a small number of founder individuals with limited genetic diversity were probably introduced recently in the country. Further longitudinal studies could help define the expansion range of this invasive tick.

## Declarations of interest

None
